# REMOTE ASSESSMENTS OF EMOTIONAL FUNCTIONING FOR INDIVIDUALS WITH DEMENTIA AND THEIR CAREGIVERS

**DOI:** 10.1093/geroni/igad104.1834

**Published:** 2023-12-21

**Authors:** Casey Brown, Kuan-Hua Chen, Julian Scheffer, Deepak Paul, Robert Levenson

**Affiliations:** Georgetown University, Washington, District of Columbia, United States; University of California, Berkeley, Berkeley, California, United States; University of California, Berkeley, Berkeley, California, United States; University of California, Berkeley, Berkeley, California, United States; University of California, Berkeley, Berkeley, California, United States

## Abstract

Emotional impairments are a common symptom of neurodegenerative disorders that can be devastating for persons with the dementia (PWDs) and family members who provide care (i.e., caregivers). Emotional functioning can be well characterized and quantified using laboratory assessments of behavioral, physiological, and self-reported responses to emotional stimuli. However, continuous declines in functioning make it especially difficult for PWDs and caregivers to visit a research laboratory repeatedly over time. In order to understand changes in emotional functioning across time and their impact on PWDs and caregivers longitudinally, there is a need for technology to support remote assessments of emotional functioning. We developed a portable version of laboratory assessments—Laboratory in a Suitcase Assessment (LISA)—to collect data from PWDs and their caregivers remotely in their homes. In a shippable suitcase, LISA provides the necessary equipment for: (a) presenting emotional stimuli via laptop screen and headphones; (b) obtaining audio and video recordings of participants’ behavior; (c) obtaining continuous ratings of emotional experience via a rating dial; (d) measuring cardiovascular, electrodermal, and somatic responses from PWDs and caregivers via wearable devices; (e) synchronizing behavior and physiology; and (f) maintaining two-way communication with participants via Zoom. We present high quality physiological, behavioral, and self-report data collected using LISA with 17 PWD-caregiver dyads. Data collected with LISA are comparable to data collected in the laboratory, indicating that LISA could enable researchers to continue data collection further into the course of disease when travel to in-person laboratory sessions would no longer be feasible.

